# Blood metal ions after hybrid metal-on-polyethylene Exeter−Trident total hip replacement

**DOI:** 10.1007/s10195-015-0369-4

**Published:** 2015-07-24

**Authors:** Rohit Singh, Gopikanthan Manoharan, Pete Craig, Simon Collier, Phillip Shaylor, Ashok Sinha

**Affiliations:** Robert Jones Orthopaedic Hospital, Oswestry, UK; Mid Staffordshire foundation trust, Stafford, England, UK

**Keywords:** Hip, Metal, Ions, Exeter, Hybrid, Trident

## Abstract

**Background:**

Metal-on-metal total hip replacements (THRs) with large femoral heads have been associated with elevated levels of cobalt (Co) and chromium (Cr), which have been attributed to high levels of wear at the articular surface. Our unit recently published data showing a significant increase in the mean levels of Co ions in patients with a 36-mm diameter femoral head with the metal-on-polyethylene Trident−Accolade system. The aim of this study is to assess the levels of Co and Cr in the Exeter−Trident hybrid system, as similar findings would raise concern over the V40 taper trunnion.

**Materials and methods:**

The study included 83 patients (45 male and 38 female with a mean age of 75.6 years) who received Exeter−Trident hybrid metal-on-polyethylene THRs. The patients were then divided into two groups according to the diameter of the femoral head used—38 patients in the 28-mm group (control), and 45 in the 36-mm (experimental) group. Serum levels of blood Co and Cr were analysed for all recruited patients.

**Results:**

In the control group (28-mm femoral head) all Co and Cr values were normal (under the abnormal threshold), as were the experimental group (36-mm femoral head). The data values were below <10 nmol and <40 nmol for Co and Cr, respectively.

**Conclusion:**

Since the National Joint Registry (NJR) states that the Exeter femoral stem is the commonest cemented femoral stem prosthesis used in the UK, we found it imperative that these results are documented given the corresponding findings in the Trident−Accolade system in our previous study. This study provides relative reassurance that the issue does not lie with the V40 taper trunnion, but raises suspicion that the issue may be with the titanium Accolade stem with large diameter femoral heads.

*Level of evidence *III.

## Introduction

Metal-on-metal (MoM) total hip replacements (THRs) with large femoral heads have been associated with elevated levels of cobalt (Co) and chromium (Cr) [1–7]. These elevated levels have been previously attributed to high levels of wear at the articular surface which have been linked to a causative factor in pseudotumour formation [8–12]. Other complications associated with increased metal ions include metal hypersensitivity, cardiomyopathy, aseptic lymphocyte-dominated vasculitis-associated lesions, and tubular necrosis [1–7]. There has been recent evidence in the literature on the possibility of the trunnion as a potential source of metal ions; however, much of this evidence is received from retrieval analysis of large head MoM THRs [13, 14].

All patients with large head MoM THRs had levels of Co and Cr ions measured, as advised by the Medicines and Healthcare products Regulatory Agency (MHRA) [15]. If these levels were above seven parts per billion (ppb; equivalent to Co 119 nmol/l and Cr 134.5 nmol/l) on two samples, collected 3 months apart, then clinical findings along with cross-sectional imaging should be used to consider revision surgery [15].

Co is a constituent of Vitamin B12 (cobalamin), a co-factor in haemoglobin metabolism [16] and Cr regulates the action of insulin, and both are important for general health. The pathological effects that have been reported in patients with raised levels of these ions following THR include visual disturbance, neurological impairment, auditory symptoms, cardiomyopathy, deep vein thrombosis, and pseudotumours [17–21]. There is a risk of contamination during the separation process when measuring levels of Co and Cr in serum of plasma. Therefore, whole blood collected in ethylenediaminetetraacetic acid or heparin is used [18].

In the tenth Annual Report of the National Joint Registry (NJR) for England and Wales, approximately 51 % of primary femoral stems in THRs in 2013 were reported as cemented fixation using stainless steel prosthesis. There has also been a steady rise in the use of large diameter (36 mm) femoral heads, from approximately 5 % of all heads to >20 % over the past 10 years [22]. This increase in the use of large diameter heads reflects the literature which states that larger heads reduce the rate of THR dislocation [23, 24]. In our unit, hybrid THR is undertaken using the Exeter−Trident system (Stryker Orthopaedics). Nationally, the Exeter femoral stem is the most commonly used cemented femoral stem in the UK. It is a polished, collarless tapered stem comprised of ‘Orthinox’, a proprietary stainless steel, which is issued with a V40 taper trunnion. The Trident acetabular component is a two-piece component with a hydroxyapatite-coated titanium alloy shell which is manufactured for press-fit following line-to-line reaming. The system utilises a unique locking mechanism providing a secure interface between the polyethylene (or ceramic) insert and the metallic shell [25].

There is little in the literature about the incidence of metallosis as a result of head–trunnion wear following metal-on-polyethylene (MoP) THR. Corrosion has been reported previously at metal junctions in THRs, and cases of pseudotumour formation attributed to metallosis in uncemented MoP THRs [26, 27].

Our unit has recently published data on a series of 69 patients who received uncemented Trident−Accolade metal on polyethylene THRs using 28- and 36-mm heads. This study showed a significant increase in the mean levels of Co ions in the blood of those with a 36-mm diameter femoral head compared to those with a 28-mm diameter head. The levels of Cr in the blood were normal in all patients. The clinical significance of this study to our unit was the suspension of use of 36-mm femoral heads for the Trident-Accolade system. The stimulus for carrying out that study was a case of severe pain following Trident−Accolade THR with a large head. This pain was secondary to severe corrosion at the head−trunnion interface leading to damage at the abductor insertion on the greater trochanter [28]. We then needed to see if this issue of raised metal ions particularly affects this combination of components in other commonly used prosthesis, such as the Exeter cemented femoral stem with the Trident acetabular cup, as this would imply an issue with V40 stem [28].

There has been no study of our magnitude directly comparing the levels of metal ions in the blood in small and large Exeter−Trident hybrid system MoP THRs. The aim of this study is to assess the levels of Co and Cr in the Exeter−Trident hybrid system as a follow-up study to see if there is a similar effect of raised metal ions using a combination of different head sizes with the V40 taper trunnion as seen in the Trident−Accolade THR.

## Materials and methods

All patients who underwent Exeter−Trident hybrid MoP THRs in 2009 and 2010 were identified using the departmental database. Two patients had died and were excluding leaving 97 patients who were contacted by letter, which explained the purpose of the study and requested their participation. This required them to undergo blood Co and Cr ion level measurements . A total of 83 patients agreed to take part in our study. All 83 patients had undergone routine follow-up and all had satisfactory outcome at the last routine clinical review, which was confirmed by validated outcome measures. Ethical approval was obtained and implied informed consent was completed by all patients prior to enrolment.

Enclosed with the initial recruitment letter was an information leaflet regarding the blood test that was produced by the laboratory, and a pre-filled clinical chemistry request form [29].

The 83 patients involved in our study were separated into two groups according to the diameter of the femoral head which was used. A 28-mm head was used in 38 patients (twenty male and eighteen female with a mean age of 76.7 years), a 36-mm head in 45 (25 male and 20 female with a mean age 74.5 years). The patients with a 28-mm head were considered as a control group as we did not expect the levels of metal ions in their blood to be raised, as our previous study with the Trident−Accolade THR found the levels for this head size to be normal. Therefore, the patients with 36-mm femoral heads were considered the experimental group.

Venesection was performed either at our hospital or in primary care. The samples were analysed at an external (clinical pathology accredited) laboratory [28]. The patients treated in 2011 and 2012 were chosen for investigation as it was felt this was a sufficient period of time for the ‘wearing-in’ of the prosthesis. The measurement of the levels of metal ions in the blood occurred at a mean of 32 months after the THR.

Results were accessed from the hospital’s electronic record system and tabulated using an Excel spreadsheet (Microsoft, Redmond, WA, USA). Abnormal results were defined as Co levels >10 nmol/l (0.59 ppb) and Cr levels >40 nmol/l (>2.07 ppb), with normal ranges for adults without THR being below these thresholds. It should be noted that the results produced by the laboratory did not state the exact value if the result was <10 nmol/l and <40 mmol/l for Co and Cr, respectively.

To our knowledge the exact mechanism of wear at the trunnion has yet to be fully determined. It is likely that it is a combination of both frictional wear and corrosion. Certain factors such as the neck offset, head length and outer diameter of the acetabular components, all of which influence the lever arm and frictional torque applied to the trunnion, would benefit from their control. This is a limitation of our study.

The data was analysed using the SPSS software v20 (IBM, Armonk, NY, USA).

## Results

In the control group (28-mm femoral head) all Co and Cr values were normal (under the abnormal threshold), as were the experimental group (36-mm femoral head) group (see Tables 1, 2).

**Table 1:** Mean and medium blood ion levels of cobalt

	28-mm femoral head	36-mm femoral head
Mean cobalt level (range) nmol/l	<10	<10
Median cobalt level (range) nmol/l	<10	<10

**Table 2:** Mean and medium blood ion levels of chromium

	28-mm femoral head	36-mm femoral head
Mean chromium level (range) nmol/l	<40	<40
Median chromium level (range) nmol/l	<40	<40

## Discussion

In this small series, patients with a 36-mm diameter modular femoral head following an Exeter−Trident hybrid MoP THR did not have higher mean levels of Co than those with 28-mm heads. We found no increase in the mean levels of Cr ions in the blood of patients with an increased diameter femoral head. In fact, the values for both groups were within the normal range for both Co and Cr.

It has been documented in the literature that there is an issue of wear at the interface between the head and trunnion which has been associated with large head MoM THRs [30]. There is a potential source of metal ion release in all metal modular junctions, which means that it is unlikely that the bearing surface is the only contributor [31].

Certain factors that have been shown to increase polyethylene wear such as acetabular inclination angles of >45° and failure to restore femoral offset were not considered in our study [31–33]. Factors that have been previously shown to affect metal ion levels in patients with hip resurfacing and MOM THR such as gender and activity level were not controlled in our study [34].

The findings from the recent previous study carried out at our unit showed increased Co levels in patients with a 36-mm head in comparison to patients with a 28-mm head in Trident-Accolade MoP THRs [28]. These findings changed the practice in our unit with the cessation of usage of 36-mm femoral heads for that specific prosthesis. These findings also raised concern as to whether there was an issue with the V40 taper trunnion with larger heads. The findings from our current study provide relative reassurance that there is no issue with the V40 taper trunnion in the Exeter orthinox prosthesis [28]. It would seem that the results from both our studies suggest that the issue may lie with the titanium femoral stem in the Accolade system as opposed to the V40 taper trunnion, as the same trunnion with the orthinox (stainless steel proprietary) Exeter stem caused no increase in metal ion level regardless of size of the femoral head. We intentionally ensured that all the Exeter femoral stems used in our study were hybrid systems with the Trident acetabular cup and can therefore certify as much interrelation between the two studies as possible. These results have been shared with the manufacturer.

A previous randomised blinded clinical trial was carried out to assess polyethylene versus metal bearing surfaces in THR. This study included forty-one patients with identical femoral and acetabular components. Erythrocyte Co and Cr levels were measured and were significantly higher in patients with MOM articulations, with an average of 7.9-fold increase in erythrocyte Co and a 2.3-fold increase in erythrocyte Cr [35]. However, there were still detectable levels with the polyethylene inserts.

The effect of taper design on trunnionosis has been extensively evaluated. A comprehensive study looked at all hip prosthesis with a 28-mm plus zero length head over fourteen years with MoP implants giving a total of forty-four sets of retrieved implants, with six different taper designs. The Goldberg scale was used to score fretting and corrosion. This study showed no difference in patient age, body mass index, or length of implantation with regard to the trunnionosis. However, Taper design had a significant effect on corrosion at the base of the trunnion [36].

The results of this study are consistent with another study which only looked at twenty patients one year after surgery with the Exeter V40 stem with a variety of acetabular components. Whole blood Cr levels were within normal limits and only one patient exhibited mild elevation of serum Co [37]. The study carried out at our unit had a much larger series of patients and only looked at one acetabular component.

The clinical significance of the findings of our study remain uncertain, but as the NJR states that the Exeter femoral stem is the commonest cemented femoral stem prosthesis used in the UK, we found it imperative that these results are documented given the corresponding findings in the Trident−Accolade system in our previous study.
